# “Derived Multiple Allogeneic Protein Paracrine Signaling (d-MAPPS)” Enhances T Cell-Driven Immune Response to Murine Mammary Carcinoma

**DOI:** 10.1155/2022/3655595

**Published:** 2022-06-15

**Authors:** Carl Randall Harrell, Dragica Pavlovic, Dragana Miloradovic, Milica Dimitrijevic Stojanovic, Valentin Djonov, Vladislav Volarevic

**Affiliations:** ^1^Regenerative Processing Plant, LLC, 34176 US Highway 19 N Palm Harbor, FL 34684, USA; ^2^Department of Genetics, Faculty of Medical Sciences, University of Kragujevac, 69 Svetozar Markovic Street, 34000 Kragujevac, Serbia; ^3^Department of Pathology, Faculty of Medical Sciences, University of Kragujevac, 69 Svetozar Markovic Street, 34000 Kragujevac, Serbia; ^4^Institute of Anatomy, University of Bern, Baltzerstrasse 2, 3012 Bern, Switzerland; ^5^Department of Microbiology and Immunology, Faculty of Medical Sciences, University of Kragujevac, 69 Svetozar Markovic Street, 34000 Kragujevac, Serbia

## Abstract

Breast cancer is considered refractory to immunotherapy. Accordingly, there is an urgent need for the therapeutic use of new immunostimulatory agents which would enhance antitumor immune response against breast cancer cells. “Derived Multiple Allogeneic Protein Paracrine Signaling (d-MAPPS)” is a biological product whose activity is based on chemokines and cytokines that modulate homing and phenotype of immune cells. d-MAPPS contains high concentration of dendritic cell (DC) and T cell-attracting chemokine CXCL16 and potent T cell-activating cytokine IL-27 which enhance DC:T cell cross-talk in inflamed tissues. Herewith, we used 4T1 murine model of breast cancer to analyze d-MAPPS-dependent enhancement of T cell-driven antitumor immunity. 4T1+d-MAPPS-treated mice showed delayed mammary tumor appearance compared to 4T1+saline-treated animals. d-MAPPS significantly reduced tumor weight and volume and improved survival of 4T1-treated mice. Significantly increased concentration of CXCL16, IL-27, IFN-*γ*, and IL-17 and decreased concentration of immunosuppressive TGF-*β* and IL-10 were measured in serum samples and tumor tissues of 4T1+d-MAPPS-treated mice. d-MAPPS enhanced production of IL-12 and increased expression of MHC class II and costimulatory molecules on tumor-infiltrated DC, significantly improving their antigen-presenting properties. d-MAPPS in CXCL16-dependent manner promoted recruitment of antitumorigenic IFN-*γ*/IL-17-producing CD4+Th1/Th17 cells and in IL-27-dependent manner induced expansion of tumoricidal CD178+granzyme B-expressing CD8+CTLs and inhibited generation of tolerogenic DC, IL-10, and TGF-*β*-producing FoxP3-expressing T regulatory cells. In summing up, d-MAPPS, in CXL16- and IL-27-dependent manner, enhanced T cell-driven antitumor immune response and suppressed breast cancer growth in experimental mice.

## 1. Introduction

Converting the immune system into the destructive power against malignant cells provides the possibility of long-term clinical benefits for cancer patients [1]. T cell-driven, tumor-specific immune response is initiated by tumor-infiltrating dendritic cells (DCs) which capture tumor antigens expressed or released from cancer cells [2]. Activated DCs process and present tumor antigens to the naïve CD4+ and CD8+ T cells which reside in the tumor draining lymph nodes [1, 2]. Interactions between DC-expressing molecules (major histocompatibility complex (MHC) proteins loaded with tumor antigens and costimulatory molecules (CD80, CD86)) with T cell-expressing receptor (TCR), coreceptors (CD4, CD8), and CD28 are necessary for optimal activation of T lymphocytes [2]. Therefore, increased presence of tumor-infiltrated CD80 and CD86-expressing DCs ensures optimal and successful generation of effector, tumoricidal T cells which would, upon activation, infiltrate the tumor tissue to eliminate cancer cells [1]. In line with these findings, a large number of experimental and clinical studies demonstrated therapeutic potential of DC-modulating agents and T cell agonists in the cancer immunotherapy [1–3]. However, breast cancer is considered immunologically quiescent and refractory to immune cell-based treatment [1]. Breast cancer cells downregulate synthesis and expression of neoantigens which prevent optimal activation of tumor-infiltrated DCs [4]. Additionally, breast cancer cells produce immunosuppressive molecules (transforming growth factor beta (TGF-*β*) and interleukin- (IL-) 10) which induce tolerogenic phenotype in DCs, macrophages, and T cells [4, 5]. Increased number of tumor-infiltrated tolerogenic DCs alternatively activated M2 macrophages and CD4+FoxP3-expressing T regulatory cells (Tregs) create immunosuppressive milieu in the breast cancer microenvironment which results in the inhibition of tumoricidal immune cells and leads to the uncontrolled tumor growth and progression [5].

Accordingly, there is an urgent need for the development and therapeutic use of new immunostimulatory agent which could increase antigen-presenting properties of tumor-infiltrated DCs, promote activation and proliferation of tumoricidal T cells, and, at the same time, suppress generation and expansion of immunosuppressive immune cells in breast cancer patients [1, 2]. In line with these findings, we recently developed the “Derived Multiple Allogeneic Protein Paracrine Signaling (d-MAPPS),” a biological product capable to modulate homing, phenotype, and function of immune cells [6]. d-MAPPS contains high concentration of DC and T cell-attracting chemokine CXCL16 and potent T cell-activating cytokine IL-27 which enhance DC:T cell cross-talk in inflamed and injured tissues [6–9]. Herewith, we used a murine model of breast cancer to analyze therapeutic potential of d-MAPPS in the generation and enhancement of T cell-driven antitumor immunity.

## 2. Material and Methods

### 2.1. Experimental Animals

Eight- to ten-week-old female BALB/c mice from animal facility of the Faculty of Medical Sciences, University of Kragujevac, Serbia, were used. All experiments were performed in accordance with the Guide for the Care and Use of Laboratory Animals (National Institutes of Health publication 86-23, 1985 revision) and were approved by the Animal Ethical Review Board of the Faculty of Medical Sciences, University of Kragujevac, Serbia.

### 2.2. Study Protocol

Animals were randomly divided into control and experimental groups (*n* = 16 mice per experimental group and *n* = 8 per control group). Mice from experimental groups received 5 × 10^4^ mouse breast cancer 4T1 cells (purchased from American Type Culture Collection (ATCC, USA)) into the fourth mammary fat pad [10]. Immediately after tumor induction, mice were randomly divided into two experimental groups to receive saline (0.1 mL/intraperitoneal injection (ip)/day; 4T1+saline-treated mice) or d-MAPPS (4T1+d-MAPPS; 0.1 mL/ip/day). Mice from control groups receive only saline or d-MAPPS (0.1 mL/ip/day).

### 2.3. Evaluation of Breast Cancer Growth and Progression

Thirty-six days after tumor induction, mice were sacrificed and the size of primary tumors was assessed by using electronic calipers and formula *V* = 4/3*π*∗*a*/2∗*b*/2∗*c*/2 (*a* = length, *b* = width, and *c* = thickness). Metastatic colonies in the paraffin-embedded, hematoxylin and eosin- (H&E-) stained lung, liver, and brain tissues were analyzed in blinded manner by a pathologist [10].

### 2.4. Measurement of Cytokine and Chemokine Content in Tumor Tissues and Serum Samples

Tumor homogenates were prepared according to the published protocol [11]. For the measurement of cytokine and chemokine serum concentration, blood was obtained from abdominal aorta [12]. The content of human CXCL16 and IL-27 and mouse IL-17, IFN-*γ*, IL-17, TGF-*β*, and IL-10 content in the tumor and serum samples was determined by commercial enzyme-linked immunosorbent assay (ELISA) sets (R&D Systems, Minneapolis, MN, USA) [12].

### 2.5. Flow Cytometry Analysis and Intracellular Staining of Tumor-Infiltrating Leukocytes

Tumor-infiltrating leukocytes were isolated form breast tumors and investigated for different cell surface and intracellular markers with flow cytometry [12]. For that purpose, 1 × 10^6^ isolated tumor-infiltrating leukocytes were incubated with anti-mouse F4/80, CD11c, NK1.1, CD80, CD86, I-A, granzyme B, CD178, CD4, and CD8 monoclonal antibodies conjugated with fluorescein isothiocyanate (FITC), phycoerythrin (PE), peridinin chlorophyll protein (PerCP), or allophycocyanin (APC) (BD Biosciences, San Jose, CA, USA). For intracellular cytokine staining, cells were stimulated with 50 ng/mL phorbol 12-myristate 13-acetate (PMA), 500 ng/mL ionomycin for 5 h, and GolgiStop (BD Biosciences) and then incubated in a BD fixation/permeabilization solution (BD Cytofix/Cytoperm™ Fixation/Permeabilization Kit) and washed in 1× BD Perm/Wash™ buffer. Fixed/permeabilized cells were stained for TNF-*α*, IFN-*γ*, IL-12, IL-4, IL-17, IL-10, and FoxP3 by using appropriate anti-mouse monoclonal antibodies conjugated with FITC, PE, PerCP, and APC (BD Biosciences, San Jose, CA, USA). BD Biosciences' FACSCalibur and Flowing Software were used for flow cytometry analysis [12].

### 2.6. Statistical Analyses

The data were analyzed using statistical package SPSS, version 21. Kolmogorov-Smirnov and Student *t*-test were used. All data were presented as the mean ± standard error of the mean (SEM). Values of *p* < 0.05 were considered statistically significant.

## 3. Results

d-MAPPS-treated mice showed delayed mammary tumor appearance and slower tumor growth by enhancing antitumor immune response.

**Figure 1:**
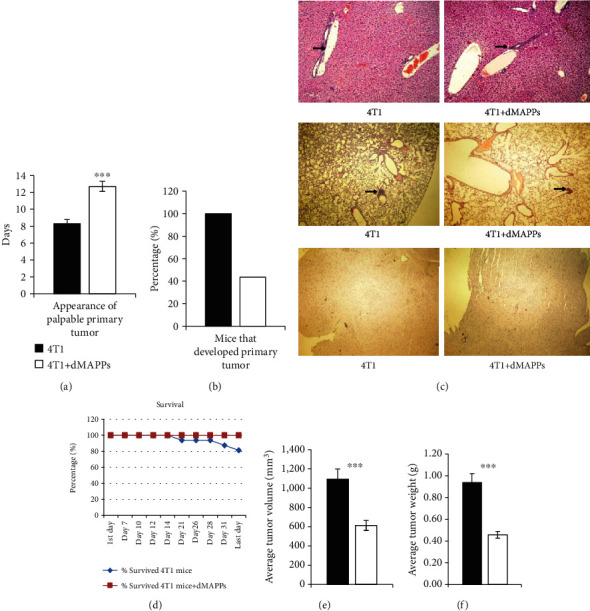
d-MAPPS delayed mammary tumor appearance and inhibited tumor growth. (a) Mean value of time period in days from inoculation of 4T1 breast cancer cells to the appearance of palpable primary tumor in 4T1+saline-treated (black bars) and 4T1+d-MAPPS-treated BALB/c mice (white bars). (b) Percentage of 4T1+saline- and 4T1+d-MAPPS-treated BALB/c mice after 36 days of follow-up. (c) Light-microscopic pictures (magnification, ×10) through liver (upper panels), lung (middle panels), and brain tissue sections (lower panels) showing metastatic colonies (black arrows). (d) Survival of 4T1+saline-treated (blue line) and 4T1+d-MAPPS-treated mice (red line). The mean value of primary tumor (e) volume and (f) weight at day 36. Data are presented as mean + /−SEM (*n* = 16 mice/group; ^∗∗∗^*p* < 0.001).

After orthotopical administration, the 4T1 cells intensively divide and rapidly form tumors which become palpable within 8-12 days [10]. As shown in Figure 1(a), d-MAPPS-treated mice show delayed mammary tumor appearance. Mean value of time period in days from inoculation of tumor cells to the appearance of palpable primary tumor in 4T1+d-MAPPS-treated mice was significantly longer than in 4T1+saline-treated animals (mean ± SEM: 12.7 days ± 0.6 versus 8.3 days ± 0.5, *p* < 0.001).

Importantly, d-MAPPS prevented development of breast cancer in majority of 4T1-treated mice. Precisely, 43.75% of 4T1+d-MAPPS-treated mice (7 out of 16) developed tumor while all (100%) of 4T1+saline-treated mice developed breast cancer (Figure 1(b)). Lung and liver metastatic colonies were determined in all 4T1+saline-treated mice and in 4T1+d-MAPPS-treated mice that developed tumors (Figure 1(c) upper and middle panels), while brain metastasis was not found either in 4T1+saline- or 4T1+d-MAPPS-treated tumor-bearing mice (Figure 1(c) lower panels). Accordingly, d-MAPPS significantly improved survival of 4T1-treated mice. While 18.75% (3 out of 16) of 4T1+saline-treated died due to the tumor progression and dissemination (Figure 1(d)), all of 4T1+d-MAPPS-treated mice survived till the end of experiment (day 36). On day 36, the mean value of primary tumor volume in 4T1+saline-treated mice was significantly higher than in 4T1+d-MAPPS-treated mice (Figure 1(e); *p* < 0.001). Similarly, primary tumor weight was significantly higher in saline-treated than in d-MAPPS-treated tumor-bearing animals (Figure 1(f); *p* < 0.001).

**Figure 2:**
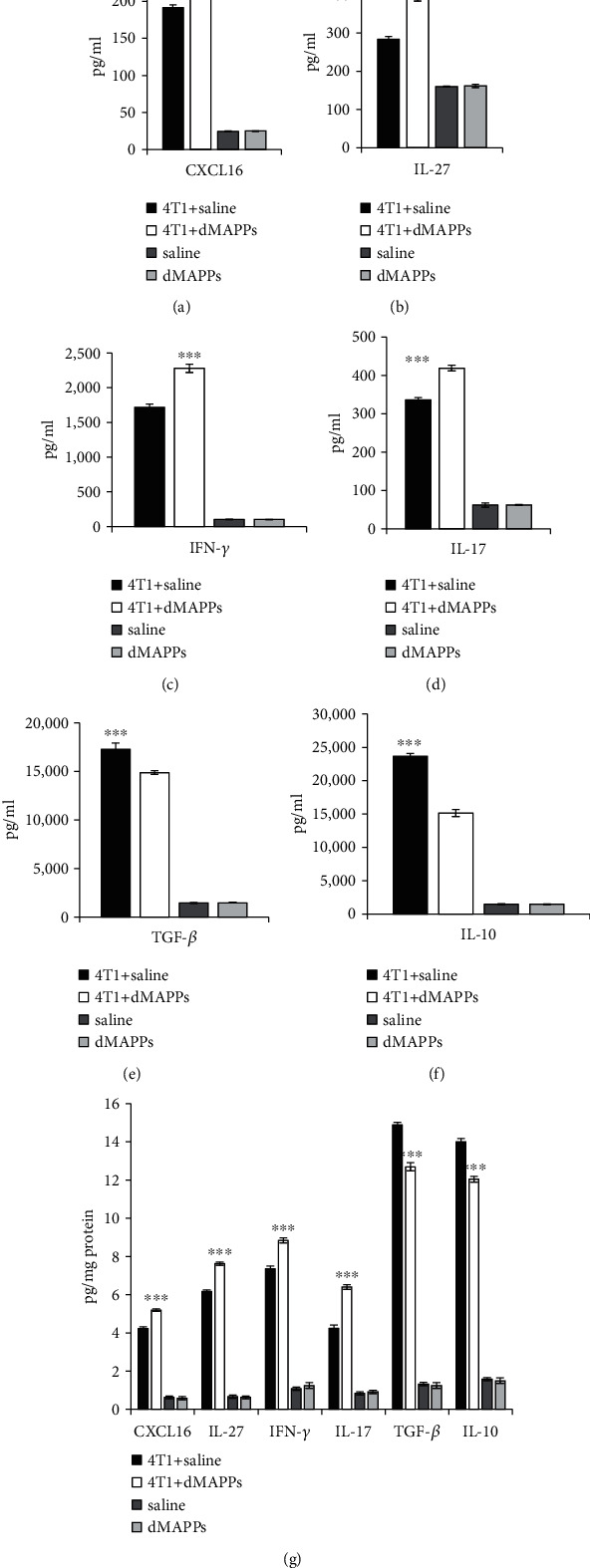
d-MAPPS enhanced concentration of antitumorigenic and reduced concentration of immunosuppressive cytokines in serum and tissue samples of tumor-bearing mice. Concentration of antitumorigenic (CXCL16, IL-27, IFN-*γ*, and IL-17) and immunosuppressive cytokines (TGF-*β* and IL-10) in (a–f) serum and (g) tumor tissue samples of BALB/c mice from experimental groups (4T1+saline-treated (black bars) and 4T1+d-MAPPS-treated animals (white bars)) and control groups (saline-treated (dark grey bars) and d-MAPPS-treated animals (light grey bars)). Data are presented as mean + /−SEM (*n* = 16 mice/experimental group and *n* = 8/control group; ^∗∗∗^*p* < 0.001).

Importantly, d-MAPPS significantly increased serum levels of antitumorigenic chemokines and cytokines CXCL16 (Figure 2(a); *p* < 0.001), IL-27 (Figure 2(b), *p* < 0.001), IFN-*γ* (Figure 2(c); *p* < 0.001), and IL-17 (Figure 2(d); *p* < 0.001) and downregulated concentration of immunosuppressive cytokines TGF-*β* (Figure 2(e); *p* < 0.001) and IL-10 (Figure 2(f); *p* < 0.001) in mice with established mammary tumors. Analogously, increased concentration of CXCL16, IL-27, IFN-*γ*, and IL-17 and decreased concentration of TGF-*β* and IL-10 were measured in the tumors of 4T1+d-MAPPS-treated mice (Figure 2(g); *p* < 0.001), indicating that modulation of antitumor immune response was mainly responsible for d-MAPPS-dependent suppression of mammary cancer growth.

### 3.1. d-MAPPS Improved Antigen-Presenting Properties of Tumor-Infiltrated Dendritic Cells

**Figure 3:**
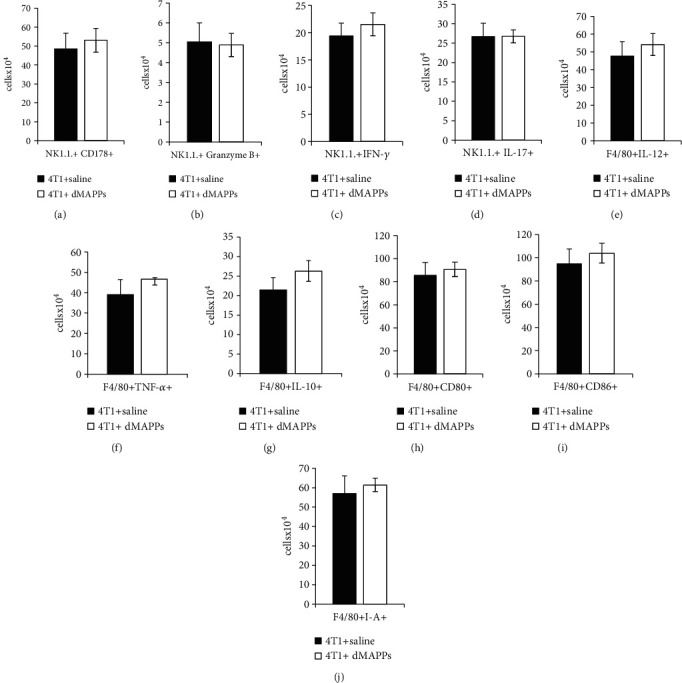
d-MAPPS did not significantly alter phenotype and function of tumor-infiltrated NK cells and macrophages. Flow cytometry analysis and intracellular staining of (a) tumor-infiltrated CD178+, (b) granzyme B+, (c) IFN-*γ*+, IL-17+ NK1.1+NK cells and (e) IL-12+, (f) TNF-*α*+, (g) IL-10+, (h) CD80+, (i) CD86+, and (j) I-A+ F4/80+ macrophages in the tumors of 4T1+saline-treated (black bars) and 4T1+d-MAPPS-treated mice (white bars). Data are presented as mean + /−SEM (*n* = 16 mice/group).

In order to dissect out the contribution of innate immunity in d-MAPPS-based modulation of breast cancer growth and progression, phenotype and function of DCs, natural killer (NK) cells, and macrophages were analyzed in the tumors of 4T1+saline- and 4T1+d-MAPPS-treated mice.

d-MAPPS did not significantly alter phenotype and function of NK cells and macrophages (Figure 3). There was no significant difference in the total number of tumor-infiltrated, cytotoxic CD178 and granzyme B-expressing, IFN-*γ* or IL-17-producing NK1.1+NK cells (Figures 3(a)–3(d)) and in the total number of IL-12-, TNF-*α*-, and IL-10-producing, CD80, CD86, and I-A-expressing F4/80+ tumor-associated macrophages (Figures 3(e)–3(j)) between 4T1+saline- and 4T1+d-MAPPS-treated mice.

**Figure 4:**
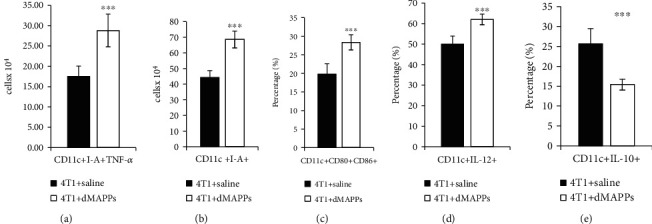
d-MAPPS improved antigen-presenting properties of tumor-infiltrated dendritic cells. Flow cytometry analysis and intracellular staining of tumor-infiltrated (a) I-A-expressing TNF-*α*-producing CD11c+, (b) I-A-expressing CD11c+, (c) CD80- and CD86-expressing CD11c+, (d) IL-12-producing CD11c+, and (e) IL-10-producing CD11c+ DCs in the tumors of 4T1+saline-treated (black bars) and 4T1+d-MAPPS-treated mice (white bars). Data are presented as mean + /−SEM (*n* = 16 mice/group; ^∗∗∗^*p* < 0.001).

Importantly, d-MAPPS significantly enhanced antigen-presenting properties of tumor-infiltrated DCs (Figure 4). Significantly higher number of DCs that express MHC class II molecule (I-A) and produce TNF-*α* (Figures 4(a) and 4(b); *p* < 0.001) and higher percentage of DCs that express costimulatory CD80 and CD86 molecules (Figure 4(c); *p* < 0.001) and produce IL-12 (Figure 4(d); *p* < 0.001) were observed in the tumors of 4T1+d-MAPPS-treated mice. Additionally, d-MAPPS reduced percentage of tolerogenic, IL-10-producing DCs in the tumors (Figure 4(e); *p* < 0.001) preventing tumor cell-driven generation of immunosuppressive microenvironment.

### 3.2. d-MAPPS Enhanced T Cell-Driven Immune Response to Murine Mammary Carcinoma

**Figure 5:**
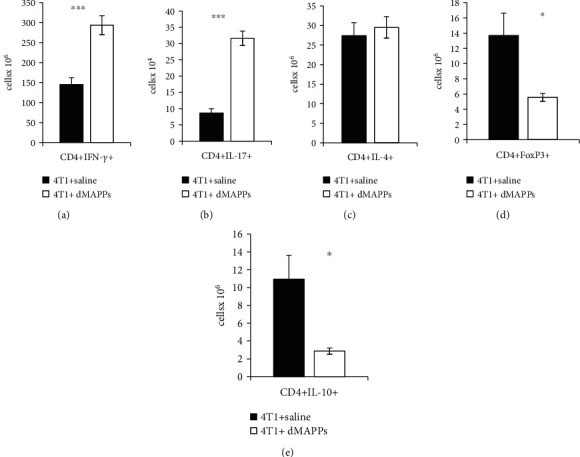
d-MAPPS increased antitumorigenic CD4+ Th1 and Th17 cells and decreased immunosuppressive T regulatory cells in the tumors of 4T1-treated mice. Total number of (a) IFN-*γ*-producing CD4+ Th1 cells, (b) IL-17-producing CD4+ Th17 cells, (c) IL-4-producing CD4+ Th2 cells, (d) IL-10-producing CD4+ T cells, and (e) FoxP3-expressing CD4+ Tregs in the tumors of 4T1+saline-treated (black bars) and 4T1+d-MAPPS-treated mice (white bars), as determined by the flow cytometry analysis and intracellular staining of tumor-infiltrated mononuclear cells. Data are presented as mean + /−SEM (*n* = 16 mice/group; ^∗^*p* < 0.05; ^∗∗∗^*p* < 0.001).

**Figure 6:**
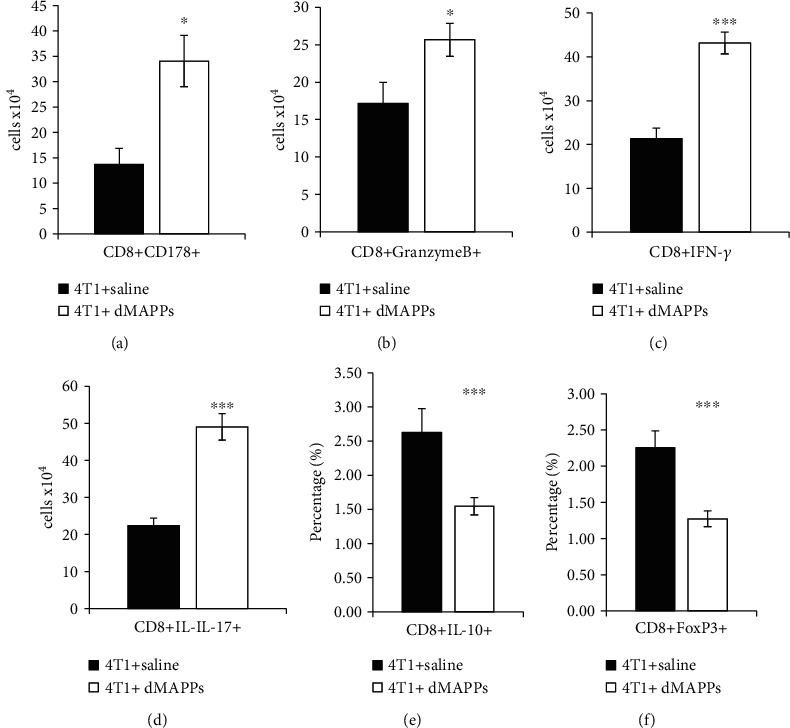
d-MAPPS increased number of tumoricidal CD8+ CTLs and decreased presence of immunosuppressive CD8+ T cells in the tumors of 4T1-treated mice. Total number of (a) tumorotoxic CD178+CD8+ CTLs, (b) granzyme B-expressing CD8+ CTLs, (c) IFN-*γ*-producing CD8+ Th1, and (d) IL-17-producing CD8+ Th17 cells and percentage of (e) immunosuppressive IL-10-producing CD8+ and (f) FoxP3-expressing CD8+ T regulatory cells in the tumors of 4T1+saline-treated (black bars) and 4T1+d-MAPPS-treated mice (white bars), as determined by the flow cytometry analysis and intracellular staining of tumor-infiltrated mononuclear cells. Data are presented as mean + /−SEM (*n* = 16 mice/group; ^∗^*p* < 0.05; ^∗∗∗^*p* < 0.001).

As a consequence of d-MAPPS-induced increased antigen-presenting activity of tumor-infiltrated DCs (Figure 4), significantly higher number of antitumorigenic CD4+Th1 and Th17 cells were observed in the mammary cancers of 4T1+d-MAPPS-treated mice (Figures 5(a)–5(c)). Total numbers of IFN-*γ*-producing Th1 and IL-17-producing Th17 cells were significantly increased in the breast tumors of 4T1+d-MAPPS-treated animals (Figures 5(a)–5(c); *p* < 0.001) while there was no significant difference in total number of CD4+IL-4+ Th2 cells between the tumors of 4T1+saline- and 4T1+d-MAPPS-treated mice (Figure 5(d)), confirming that d-MAPPS favored generation of antitumorigenic Th1 and Th17 immune response. Moreover, d-MAPPS inhibited generation of immunosuppressive phenotype in tumor-infiltrated CD4+ T cells (Figures 5(e) and 5(f)). A remarkably decreased number of FoxP3-expressing and IL-10-producing CD4+ Tregs were observed in the tumors of 4T1+d-MAPPS-treated animals compared to tumor-bearing animals that received saline (Figures 5(e) and 5(f); *p* < 0.05).

d-MAPPS significantly increased the presence of tumor-infiltrated CD178-expressing and granzyme B-producing cytotoxic CD8+ T cells (CTLs; Figures 6(a) and 6(b); *p* < 0.001). Additionally, d-MAPPS favored activation and expansion of tumoricidal IFN-*γ*-producing and IL-17-producing CD8+ CTLs (Figures 6(c) and 6(d); *p* < 0.001) and inhibited generation of immunosuppressive and protumorigenic IL-10-producing and FoxP3-expressing CD8+ T cells (Figures 6(e) and 6(f); *p* < 0.001).

## 4. Discussion

The cross-talk between activated DCs and antigen-specific CD4+ T helper and CD8+ cytotoxic T lymphocytes is crucially responsible for the generation of cellular immunity to tumor antigens [1]. During long-term tumor growth and progression, high levels of tumor antigens and the inflammatory environment lead to the persistent activation of antigen-specific T cell receptors which results in functional exhaustion and inefficacy of antigen-specific T cells [13]. Accordingly, recruitment of new, nonexhausted T cells in the tumors is crucially responsible for the generation of effective T cell-driven antitumor immunity [2, 13].

d-MAPPS contains high concentration of immunostimulatory molecules (CXCL16 and IL-27) which enhance DC-dependent generation of effector T cells, promote their recruitment in the tumors, and increase their tumoricidal potential [6–9]. Upon capture of tumor antigens, antigen-presenting DCs produce CXCL16 that recruit CXCR6-expressing effector T cells in tumor tissue [8]. Additionally, CXC16 activates tissue-resident memory T cells and is considered critical for sustained T cell-mediated tumor protection [1, 8]. Continuous administration of d-MAPPS increased concentration of CXCL16 in the tumor microenvironment (Figure 2(g)) and attracted tumoricidal, perforin, and granzyme-expressing CD8+CTLs (Figures 6(a) and 6(b)), CD4+Th1, and Th17 lymphocytes in the tumors of 4T1+d-MAPPS-treated mice (Figures 5(a) and 5(b)).

DC-derived TNF-*α* and IL-12 generate Th1 and Th17 phenotype in T cells by inducing expression of transcriptional factors T-bet and ROR-yT in STAT-1- and STAT-3-dependent manner [14]. d-MAPPS contains high concentration of IL-27 which activates STAT-1 and STAT-3 signaling pathways in naïve T cells, facilitating DC-dependent, IL-12- and TNF-*α*-driven generation of Th1 and Th17 cells [15, 16]. Accordingly, continuous administration of d-MAPPS increased concentration of IL-27 in the microenvironment of breast cancers (Figure 2(g)) which, in accordance with increased presence of tumor-infiltrated TNF-*α* and IL-12-producing DCs (Figures 4(a) and 4(d)), resulted in the expansion of IFN-*γ*- and IL-17-producing CD4+ and CD8+ Th1 and Th17 cells in the tumors of 4T1+d-MAPPS-treated mice (Figures 5(a) and 5(b) and Figures 6(c) and 6(d)). Th1 cells directly kill malignant cells via release of TNF-*α* which activates TNF-related apoptosis-inducing ligand (TRAIL) on the tumor cell surface [17]. Additionally, Th1 cell-derived IFN-*γ* induces enhanced production of tumorotoxic nitric oxide and TNF-*α* in tumor-infiltrated macrophages while Th17 cell-sourced IL-17 stimulate production of CXCL16 in tumor DCs, enabling increased recruitment of tumoricidal CCR6-expressing CD8+CTLs [17, 18].

In addition to its effects on the generation of CD4+ Th1 and Th17 cells, IL-27 induces enhanced synthesis of the transcription factors T-bet and eomesodermin in naïve CD8+ T lymphocytes, enabling their conversion in effector CD8+ CTLs [19]. Moreover, IL-27 enhances production of proapoptotic molecules (granzyme B and perforin) in effector CD8+ CTLs, enhancing their cytotoxicity [19]. Accordingly, results obtained in murine models of plasmacytoma, melanoma, and neuroblastoma showed that both systemic and intratumorally delivered IL-27 enhanced presence of CD8+ CTLs in tumor microenvironment which resulted in the tumor regression [19].

In tumor-bearing animals, IL-27 downregulated the expression of FoxP3 in CD4+ and CD8+ T cells and prevented generation and expansion of Tregs, immunosuppressive T cells which inhibited antitumor immunity in IL-10- and TGF-*β*-dependent manner [19, 20]. In line with these findings, increased levels of IL-27, found in serum samples and tumor tissues of 4T1+d-MAPPS-treated mice (Figures 2(b) and 2(g)), were accompanied with reduced number of tumor-infiltrated IL-10- and TGF-*β*-producing, FoxP3-expressing Tregs (Figures 5(e) and 5(f)) and with downregulated serum levels of IL-10 and TGF-*β* (Figures 2(e) and 2(f)). Accordingly, we believe that d-MAPPS containing IL-27 was mainly responsible for decreased presence of immunosuppressive Tregs in 4T1+d-MAPPS-treated mice.

Although IL-27 efficiently suppressed tumor growth and progression in tumor-bearing animals, clinical use of recombinant IL-27 is limited by the fact that continuous delivery of IL-27 may generate strong systemic T cell-driven immune response which could result in the development of life-threatening cytokine storm [19]. d-MAPPS is more available for clinical application than recombinant IL-27 since it contains several immunoregulatory molecules (interleukin 1 receptor antagonist, soluble TNF-*α* receptors, and growth-related oncogene gamma) which may limit IL-27-driven overactivation of T cells and prevent generation of cytokine storm [6, 7].

Despite the fact that d-MAPPS prevented tumor development in majority of 4T1-treated animals and remarkably inhibited growth of primary breast cancer in mice that developed tumor (Figures 1(a) and 1(b)), it should be noted that it did not manage to significantly reduce number and size of liver and lung metastatic colonies in tumor-bearing animals (Figure 1(c)). In this study, d-MAPPS-induced effects on phenotype and function of tumor-infiltrating immune cells were demonstrated. Therapeutic potential of d-MAPPS in modulation of systemic antitumor immune response, including d-MAPPS-induced changes in number and effector function of circulating leukocytes, should be examined in upcoming studies which will investigate efficacy of d-MAPPS in the suppression of metastatic breast cancer. Since majority of d-MAPPS-based antitumor effects relied on the activity of d-MAPPS containing IL-27 and IL-27-based antitumor therapy had significant synergy with checkpoint inhibitors that prevented excessive exhaustion of tumoricidal T cells [21, 22], we believe that checkpoint inhibitors will significantly enhance systemic antitumor effects of d-MAPPS. Accordingly, upcoming studies should explore synergistic effects of d-MAPPS and checkpoint inhibitors in the suppression of breast cancer dissemination.

## 5. Conclusions

In summing up, d-MAPPS augmented T cell-driven immune response to murine mammary carcinoma by enhancing DC-based generation of Th1 and Th17 cells and by increasing cytotoxicity of CD8+ CTLs in CXCL16- and IL-27-dependent manner. d-MAPPS could be considered as new immunostimulatory biological agent which antitumor efficacy should be further explored in upcoming immune-oncology studies.

## Data Availability

The data used to support the findings of this study are included within the article.
